# Control of a Clonal Outbreak of Multidrug-Resistant *Acinetobacter baumannii* in a Hospital of the Basque Country after the Introduction of Environmental Cleaning Led by the Systematic Sampling from Environmental Objects

**DOI:** 10.1155/2013/582831

**Published:** 2013-12-30

**Authors:** Jesús Delgado Naranjo, José Ignacio Villate Navarro, Mercedes Sota Busselo, Alberto Martínez Ruíz, José María Hernández Hernández, María Pilar Torres Garmendia, María Isabel Urcelay López

**Affiliations:** ^1^Service of Preventive Medicine, University Hospital of Cruces, Basque Health Service OSAKIDETZA, 48903 Barakaldo, Bizkaia, Spain; ^2^Department of Preventive Medicine and Public Health, Faculty of Medicine, University of the Basque Country, 48940 Leioa, Bizkaia, Spain; ^3^Laboratory of Microbiology, University Hospital of Cruces, Basque Health Service OSAKIDETZA, 48903 Barakaldo, Bizkaia, Spain; ^4^Service of Anaesthesia and Reanimation, University Hospital of Cruces, Basque Health Service OSAKIDETZA, 48903 Barakaldo, Bizkaia, Spain

## Abstract

*Background*. Between July 2009 and September 2010, an outbreak of multidrug-resistant (MDR) *Acinetobacter baumannii* was detected in one critical care unit of a tertiary hospital in the Basque Country, involving 49 infected and 16 colonized patients. The aim was to evaluate the impact of environmental cleaning and systematic sampling from environmental objects on the risk of infection by MDR *A. baumannii*. *Methods*. After systematic sampling from environmental objects and molecular typing of all new MDR *A. baumannii* strains from patients and environmental isolates, we analyzed the correlation (Pearson's *r*) between new infected cases and positive environmental samples. The risk ratio (RR) of infection was estimated with Poisson regression. *Results*. The risk increased significantly with the number of positive samples in common areas (RR = 1.40; 95%CI = 0.99–1.94) and positive samples in boxes (RR = 1.19; 95%CI = 1.01–1.40). The number of cases also positively correlated with positive samples in boxes (*r* = 0.50; *P* < 0.05) and common areas (*r* = 0.29; *P* = 0.18). *Conclusion.* Once conventional measures have failed, environmental cleaning, guided by systematic sampling from environmental objects, provided the objective risk reduction of new cases and enabled the full control of the outbreak.

## 1. Introduction

*Acinetobacter baumannii* is a ubiquitous, gram-negative and nonfermenting bacillus that can cause both community and healthcare-associated infections due to its antimicrobial resistance, thereby leading to pan-drug resistance and causing large tertiary intrahospital outbreaks, often involving multiple facilities [1]. The multidrug-resistant (MDR) *A. baumannii* is an opportunistic pathogen that can cause health care-associated infections due to its antimicrobial resistance, resistance to desiccation and capability of surviving on the surfaces of critical areas [2]. The major risk factors for infection include invasive procedures, such as the use of mechanical ventilation, central venous or urinary catheters, and broad-spectrum antimicrobials [3].

In this paper we describe an outbreak of MDR *A. baumannii* in one of the critical care units of our tertiary hospital in the Basque Country from July 2009 to September 2010. The goal of the study was to analyze the impact of the environmental factors and cleaning measurements taken over the evolution of the outbreak and to interpret these results from an epidemiological point of view.

## 2. Materials and Methods

### 2.1. Setting

The critical care area where the outbreak arose is subdivided into three units (A1, A2, and A3), with a total of 33 beds, all of which were suited in individual rooms, except for a bay area (A3) with 6 beds. In the three units there was a common area containing the nurse station desk and the automated medication dispensing system.

The outbreak arose in A2, but it spread rapidly to the other wards (A1 and A3). The occupation index of the critical care unit was approximately 100% when the index case was detected. During the outbreak a new bay area was enabled (R-0). This provisional unit was used to accept patients while the critical care unit was being thoroughly cleaned.

### 2.2. Case Definition

A case was defined as a patient with at least one clinical or screening sample that was positive for a MDR* A. baumannii*. We considered the infected cases to include those patients who had positive laboratory tests with clinical symptomatology. On the other hand, we consider as carriers to those patients who were colonized with *A. baumannii*, when screening samples were positive without clinical manifestations of disease (colonized patients). The multiple resistance of *A. baumannii* was defined as the nonsusceptibility to the carbapenems (intermediate or resistant), other *β*-lactams, the fluoroquinolones, or amikacin [4].

### 2.3. Systematic Process of Environmental Sampling

A systematic process of environmental sampling was introduced during the second part of the outbreak, between 10th and 38th epidemiological weeks of 2010. Samples for cultures were collected weekly from the surface of the objects following a weekly sampling scheme. The environmental sampling included the following: (i) the patients' direct environment, including the equipment and other surfaces belonging to the rooms and (ii) the common areas (not included in the rooms).

Environmental isolates in rooms were systematically taken from bed frames, monitors, infusion pumps, push button of automatic doors, washbasins, taps and other objects and surfaces (chairs, tables, etc.). Environmental isolates in common areas were obtained from high drawer chests, handles, counter tops, nurse station desks, automated medication dispensing systems, washbasins, taps, other objects and surfaces (chairs, tables, computers, CPUs, keyboards, etc.).

Samples for cultures were collected weekly from the surface of the objects belonging to the rooms were a new case was detected. On the other hand, samples from common areas were taken once a week, except for 10th, 13th, and 14th weeks. We took one environmental sample per mentioned object in rooms, so the total number of environmental samples was 6 for each room were a case was detected, except for 31th and 35th weeks, when the sampling was extended to other surfaces. On the other hand, we obtained one environmental sample per listed object in common areas, so the total number of environmental samples was 7 for each common area, except for 11th, 22th, 24th, 31th, and 35th weeks, when the sampling was extended to other objects and surfaces.

### 2.4. Microbiological Analysis of Environmental Samples

The environmental samples were processed using moist gauze following a modified method described previously [5]. Briefly, the sterile gauze pads, moistened with BHI broth, were used for the environmental sampling. Gauze pads were incubated with the BHI broth at 37°C for 24 hours and then inoculated in Mac Conckey agar for 24–48 hours. The identification and antimicrobial sensitivity tests were performed using the Vitek2 system. After the item sampling, all surfaces were cleaned and disinfected.

### 2.5. Microbiological Analysis of Patient Samples

The MDR *A. baumannii* was recovered from the following suspicious infected tissues or organs: the respiratory tract (including the sputum, bronchial aspirate and bronchial alveolar specimens), the urinary tract, the blood, and the drainage fluid, interalia. Twice each week for the carriers study, we obtained the surface samples (from the axilla, groin, and pharynx) from all patients admitted to the critical area. The MDR *A. baumannii* was isolated in the conventional culture media, and the identification was performed using the Vitek2 system or API strips (bioMérieux, Marcy l'Etoile, France). The antimicrobial susceptibility testing was determined using disk diffusion tests or the Vitek2 system (MIC panels) as the cases.

### 2.6. Molecular Typing of Isolates

We performed the molecular typing of all new MDR *A. baumannii* strains isolated in the hospital (including one isolate per patient and the environmental isolates). The molecular typing method used was the rep-PCR DiversiLab Microbial Typing System (bioMérieux, Marcy l'Etoile, France), following the manufacturer's instructions [6]. The clonal pattern of the outbreak *A. baumannii* was determined and all *A. baumannii* isolates were compared with other *A. baumannii* clones detected in the hospital before. Results analysis was then performed with the DiversiLab software (version 2.1.66a), which uses the Pearson correlation coefficient to determine distance matrices and the unweighted pair group method with arithmetic averages (UPGMA) to create dendrograms. Reports were automatically generated including the dendrograms, electropherograms, virtual gel images, and selectable demographic fields to aid in the data interpretation.

### 2.7. Outbreak Investigation

The investigation of the outbreak was coordinated by the critical unit outbreak control committee, consisting of the Hospital Executive, the medical and nursing staff from the affected area, the Unit of Preventive Medicine and the Microbiology Laboratory. During the outbreak, the clinic and screening samples collected from the patients, as well as environmental samples, were taken according to the scheme mentioned above.

All the clinical samples were taken as part of standard care. The research was carried out in compliance with the Helsinki Declaration and complies with the general regulations concerning medical research in humans. The performed study was approved by the Clinical Research and Ethics Committee of the University Hospital of Cruces (reference number 2012/09-01).

### 2.8. Epidemiologic and Statistical Analysis

A descriptive analysis of the evolution of the outbreak was performed, representing the number of newly infected and carrier cases throughout the outbreak, and the epidemiologic curve was plotted. We studied the outbreak characteristics by estimating the proportions and their 95% confidence intervals (95%CI) for categorical variables as well as the means with their 95%CI for continuous data.

We analyzed the correlation (Pearson's *r*) between the number of new infected cases in the area and the following different variables: the number of carriers, the number of positive environmental samples in rooms and the positive environmental samples in common areas.

The risk ratio (RR) of new infected cases in the critical area was estimated by separate univariate Poisson regression models, in which the dependent variable was the number of new infected cases, and the independent variables included the number of carriers, the number of positive environmental samples in rooms, and the number of positive samples in common areas.

The temporal trend of the number of new cases in the area throughout the outbreak was analyzed with univariate Poisson regression models, in which the dependent variable was the number of new infected cases, and the independent variables included the following measurements of time: weeks (continuous data), months (continuous data) and quarters (categorical and continuous data).

All statistical analyses were performed using STATA v. 12.0 (StataCorp. 2011. Stata Statistical Software: Release 12. College Station, TX: StataCorp LP.). The analysis was considered statistically significant when *P*-value was equal to or less than 0.05.

## 3. Results

### 3.1. Outbreak Investigation

**Figure 1:**
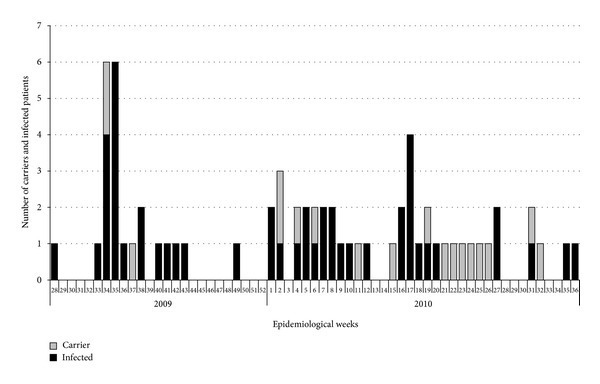
Epidemic curve expressing the incidence of cases (carriers and infected patients) involved in the *A. baumannii* outbreak over time (epidemiologic weeks). After the 36th week of 2010, no new cases were registered.

A total of 49 infected and 16 carrier patients were detected from July 2009 to September 2010, following the weekly temporal distribution represented in Figure 1. The index case of the outbreak was detected at 33th week, but the first case occurred at 28th of 2009. The outbreak consisted of two phases, with two peaks of incidence. During the first phase of the outbreak, between the 26th and 48th week of 2009, infection incidence was detected with an important incidence peak of 6 cases. During the second phase of the outbreak, between the 49th week of 2009 and 36th week of 2010, infection incidence was registered with the second peak of the outbreak in 4 cases. The last case was detected at 36th week but the critical unit outbreak control committee remained active and a further epidemiologic surveillance was made during the rest of 2010 and during 2011. In January 2011 a preliminary report was drawn up and the definitive report was established in June 2011. The epidemiologic surveillance of multidrug resistant microorganisms mandatory in the Public Health System of the Basque Country) has not detected any new case.

A total of 27 patients (41%) came into the unit after surgery, 31 (47%) were admitted from the emergency unit, and the remainder were nonsurgical patients coming from different clinic services in the hospital (Table 1). The most frequent infection site was the respiratory system (including the sputum, bronchial aspirate, and bronchial alveolar specimens), which accounted for 30 cases (46%). A total of 26 patients died, representing a mortality rate of 40% among all of the infected and colonized cases.

All cases were detected in the critical care unit (A1, A2, and A3 areas), except for one case who was diagnosed in R0, the provisional unit enabled while the other areas of the critical care unit were being cleaned.

**Figure 2:**
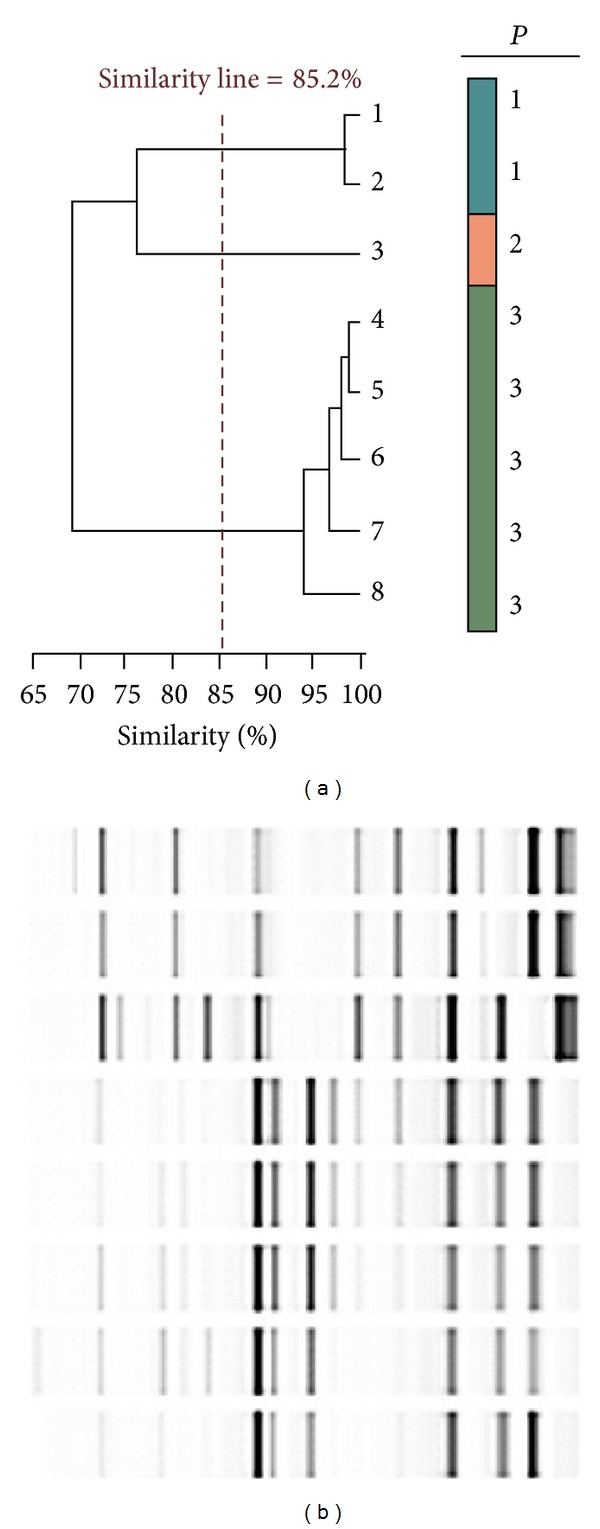
Example of molecular typing of isolates used during the outbreak. It is shown a DiversiLab system-generated dendrogram, showing the first five case outbreak *A. baumannii* isolates, compared with two other *A. baumannii* clones, detected before in the Hospital. The dendrogram shows strain clustering. The horizontal bar at the bottom left of the dendrogram indicates the percent similarity coefficient within the strains. Spacing between grid lines indicates increments of 5% similarity based on the proximity matrix results.

It was found clonal correlation among samples; indeed, a single clonal lineage of multidrug-resistant *A. baumannii* was isolated. This clonal pattern was different to *other A. baumannii* clones isolated before in our hospital (Figure 2). The antibiotic susceptibility profile of the outbreak isolates was sensitive to amikacin and colistin and showed intermediate resistance to meropenem and tigecycline as well as full resistance to ampicillin-sulbactam, piperacillin-tazobactam, imipenem, aztreonam, ciprofloxacin, levofloxacin, ceftazidime, cefepime, gentamicin, tobramycin, and cotrimoxazole.

### 3.2. Outbreak Control Measures

Both the infected and carrier patients were maintained under contact precautions following the principles of the Basque Nosocomial Infection Committee (INOZ Committee) [7], based on the international protocols [8], in combination with cohort isolation measures. The active surveillance cultures from patients were systematically monitored to allow for the early detection of asymptomatic cases. The hygienic measures were intensified; the healthcare workers were instructed on the strict adherence to hand hygiene, and special formative actions were developed to encourage the staff on the security of patients. Moreover, the presence of infection control nurses in the areas was increased.

**Figure 3:**
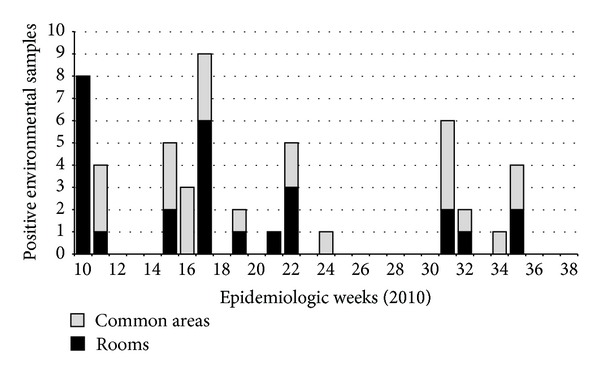
Number of positive environmental samples taken from rooms and common areas in the hospital critical care area, during de second part of the outbreak (2010). After the 36th week of 2010, all the environmental isolates were negative.

The systematic analysis of the environmental samples was introduced during the second part of the outbreak (Table 2, Figure 3), with intensified cleaning efforts, depending on the results of the microbiology analysis of these samples. Thus, the thorough cleaning of the rooms and the common areas was systematically conducted and directed, depending on the results of the environmental sampling. The cleaning procedures of both the common areas and rooms were revised and enhanced. The cleaning efforts were emphasized, especially after patient discharge. All surfaces (including the furniture, equipment and walls) were extensively cleaned and decontaminated. Care was taken not to remove any furniture or equipment from the areas prior to decontamination. The cleaning procedures were conducted by a trained cleaning staff and monitored by a Preventive Medicine nursing staff. The supplies of utility goods at the patients' bedsides were kept to a minimum. Additionally, the ward was cleaned more frequently, with particular attention paid to areas where dust was likely to gather. The surfaces were cleaned with one-time-use dusters, and different cleaning products were tested, although the disinfectant was not changed. The cleaning measures were implemented during the second phase of the outbreak and were intensified in the second and third quarters of 2010.

### 3.3. Effectiveness Analysis of Control Measures over Outbreak Development: Trends, Correlation and Regression Analysis

In Table 3, we quantify the association of different factors involved in the outbreak to the number of new infected cases, by means of the Risk Ratio (RR), estimated by univariate Poisson regression. The number of new infected patients was not associated with the number of colonized patients (RR = 1.06; 95%CI = 0.62–1.81). However, the risk of *A. baumannii* infection increased significantly with the number of positive samples in common areas (RR = 1.40; 95%CI = 0.99–1.94; *P* = 0.05) and with the number of positive samples in rooms (RR = 1.19; 95%CI = 1.01–1.40; *P* < 0.05).

A positive correlation was detected between the number of new infected cases and the positive samples from rooms (*r* = 0.50; *P* < 0.05). The number of positive results obtained from samples in common areas was positively correlated with the number of new infected cases (*r* = 0.29; *P* = 0.18). However, a negative correlation was detected between the number of infected and the number of colonized patients (*r* = −0.50; *P* < 0.05).

A temporal trend analysis of the number of new cases, using Poisson regression analysis (Table 3), showed a decreasing trend in the number of new infected cases throughout the weeks (*β* = −0.014; *P* = 0.07), months (*β* = −0.06; *P* = 0.07) and quarters (*β* = −0.16; *P* = 0.13) of the study. The risk of new infected cases in the area was 0.86 (95%CI = 0.41–1.78) during the first quarter of 2010, and the risk reduced to 0.62 (95%CI = 0.41–1.78) in the second quarter of 2010 and further dropped to 0.33 (95%CI = 0.12–0.92) during the third quarter of 2010.

## 4. Discussion

Following the resolution of this outbreak, the thorough cleaning led by the systematic testing of environmental samples from rooms and common parts in the clinic area were especially relevant. Because the outbreak consisted of two phases and because the cleaning, led by the positive cultures of environmental samples, was implemented during the second peak of the outbreak, it was possible to compare the effectiveness, in terms of the reduction of new cases, once these measures were implemented. These new steps have been especially useful during the second peak of the outbreak, after verifying that the conventional cleaning methods were insufficient to resolve the outbreak. Since these measures were implemented, a significant reduction in the number of new cases was observed. After the end of the outbreak, no new cases have been detected. These additional measures, combined with other conventional measures, such as contact isolation and screening sampling from patients, have been useful in other hospital outbreaks of *A. baumannii* [9].

The relevance of the environmental cleaning as an important tool to control the outbreak has been statistically confirmed because the number of positive cultures obtained from the environmental sampling correlated significantly with the number of new *A. baumannii* cases in the critical area (*r* = 0.50; *P* < 0.05). Moreover, the positive samples from common areas was positively correlated with the number of new infected cases (*r* = 0.29; *P* = 0.18), thereby enhancing the role of the decreasing environmental load of microorganisms in the control of our *A. baumannii* outbreak.

From an epidemiological point of view, in this outbreak, the risk of *A. baumannii* infection increased by 40% for each positive culture taken in common areas (RR = 1.40; 95%CI = 0.99–1.94; *P* = 0.05) and by 19% for each positive sample in the rooms (RR = 1.19; 95%CI = 1.01–1.40; *P* < 0.05). Consequently, the overall number of positive cultures obtained from both types of environmental samples was the variable that best predicted the new infected cases in the critical area. Thus, the environmental sampling represented an important tool to guide the extensive cleaning of the area and to test the quality of that cleaning to prevent new cases.

However, the screening control efforts, through the study of colonized patients (carriers), has had a limited role in predicting new infected cases and in the control of the outbreak. In fact, the number of colonized patients was negatively correlated with the infection incidence (*r* = −0.50; *P* < 0.05). Despite the number of the positive environmental samples increasing the risk of new cases, the number of new infected patients was not associated with the number of colonized patients (RR = 1.06; 95%CI = 0.62–1.81). One of the reasons underlying this limited role of colonization could be that we only obtained axilla, groin, and pharynx samples for the colonization study rather than rectal samples. This sampling method may show less sensitivity, but the specificity remains unaltered [10]. Nevertheless, the daily bathing of patients with chlorhexidine and the improved environmental cleaning have, in quasi-experimental studies [11, 12], shown promise in reducing the incidence of multidrug resistant microorganism colonization among Intensive Care Unit (ICU) patients [13]. Merely improving the identification of colonized patients and expanding the use of barrier precautions, at least as to the extent that we achieved, are measures that are not likely to be broadly effective [14]. If the transmission of MDR microorganisms in healthcare facilities is to be decreased substantially, an improvement in the reliable, sustainable adherence to the isolation precautions is important. This adherence may need to be complemented by interventions that reduce the density of colonization of body sites and that decrease the environmental contamination [4]. In contrast, there is no evidence that the adoption of contact precautions can provide superior effectiveness, compared with standard precautions. In fact, it is possible to control an outbreak of MDR *A. baumannii* colonization and infection in an intensive care unit (ICU) without closing the ICU or placing the patients in isolation [15]. Despite the widespread environmental contamination, the control of the outbreak was eventually achieved without resorting to the complete closure of the unit for disinfection and cleaning.

Previous studies have suggested a link between the environmental contamination of clinical areas and *A. baumannii* outbreaks. In this outbreak, as in many others, it is not possible to ascribe the control to any single factor. However, it is likely that the limitation and control of the environmental reservoirs were the major factors in controlling the described outbreak, given that the resolution of the outbreak was possible after the second peak of the outbreak, once the systematic environmental cleaning was implemented. Because *A. baumannii* spreads easily in the vicinity of infected or colonized patients and can persist in the environment for many days, even in dry conditions [10], the environmental control and cleaning appears to be valuable in the development of successful control interventions in *A. baumannii* outbreaks. In contrast, new measures have an additional pedagogic value by providing a useful strategy with which to encourage clinical staff to increase adherence to secure practices and thereby optimize outbreak security. As a result, the environmental cleaning has been emphasized in this paper and elsewhere as a major part of an effective outbreak control strategy [16, 17].

Ultimately, the end of the outbreak was made possible after the implementation of different kind of measures. As a result, the unique effects of environmental sampling remain unknown because they cannot be separated from the global effects of the conventional measures. In the described outbreak, as in many others that have been reported, it is not possible to ascribe the outbreak control to any one factor; however, it is likely that the limitation and control of the environmental reservoirs were the major factors in controlling the outbreak. It must be taken into account that we have performed a before-after study and, as in other quasi-experimental designs, in our study cause and effect must be interpreted carefully. Consequently it is not possible to establish an unbiased causality relationship between the different control measurements and MDR Acinetobacter baumannii infection, remaining unclear which control measurement had the biggest impact.

## 5. Conclusions

We were faced with a clonal outbreak caused by the multi-drug resistant *A. baumannii* in a critical care unit of a hospital of the Basque Country, involving 49 infected patients and 16 carriers. The environmental cleaning, guided by the systematic sampling from environmental objects, provided the objective risk reduction of newly infected and colonized cases. The full resolution of the outbreak was made possible due to the environmental cleaning led by the systematic sampling from environmental objects, after verifying that conventional measures, such as contact isolation and colonization study, were insufficient to resolve the outbreak. The environmental sampling represents an important tool with which to guide the extensive cleaning of at-risk-areas, and this tool can be used to evaluate the quality of the cleaning to prevent new cases and to resolve the outbreak.

## Figures and Tables

**Table 1:** Demographic and clinical characteristics of patients involved in the outbreak.

	Frequency (*n*)	Proportion (%)	95% CI (%)
Sex			
Male	45	69.23	57.70–80.75
Female	20	30.77	19.24–42.29
Age (mean, years)	65	61.15	56.71–65.60
Time in hospital (mean, days)	65	19.89	15.98–23.80
Time in unit (mean, days)	65	14.83	11.52–18.14
Critical care unit			
R0	1	1.54	−1.53–4.61
A1	23	35.38	23.44–47.32
A2	18	27.69	16.52–38.87
A3	23	35.38	23.44–47.33
Service of origin			
Surgery unit	27	41.54	29.23–53.84
Emergency	31	47.69	35.22–60.17
Other services	7	10.77	3.03–18.52
Infected/carrier status			
Infected	49	75.38	64.62–86.14
Carrier (colonized)	16	24.62	13.85–35.37
Infection site			
Respiratory system	30	46.15	33.71–58.60
Blood	9	13.85	5.22–22.47
Other sites	10	15.38	6.37–24.40
Colonized	16	24.62	13.86–35.37
Outcome			
Survive	39	60.00	47.77–72.23
Death	26	40.00	27.77–52.23

**Table 2:** Number of new cases, environmental isolates in rooms, and environmental isolates in common areas after introducing systematic sampling from environmental objects, during the second part of the outbreak.

	Epidemiological weeks (2010)																												
	10	11	12	13	14	15	16	17	18	19	20	21	22	23	24	25	26	27	28	29	30	31	32	33	34	35	36	37	38
Number of new cases																													
Infected cases	*1 *	0	*1 *	0	0	0	*2 *	*4 *	*1 *	*1 *	*1 *	0	0	0	0	0	0	*2 *	0	0	0	*1 *	0	0	0	*1 *	*1 *	0	0
Carriers	0	*1 *	0	0	0	*1 *	0	0	0	*1 *	0	*1 *	*1 *	*1 *	*1 *	*1 *	*1 *	0	0	0	0	*1 *	*1 *	0	0	0	0	0	0

Number of environmental isolates in rooms																													
Bed frames	*4 *	0	0			*1 *	0	*2 *	0	*1 *	0	*1 *	0	0	0	0	0	0				0	0			0	0		
Monitors and infusion pumps	*4 *	*1 *	0			*1 *	0	*2 *	0	0	0	0	*1 *	0	0	0	0	0				0	*1 *			0	0		
Push button of automatic doors	0	0	0			0	0	*1 *	0	0	0	0	*1 *	0	0	0	0	0				0	0			0	0		
Washbasins and taps	0	0	0			0	0	*1 *	0	0	0	0	*1 *	0	0	0	0	0				*1 *	0			0	0		
Other objects and surfaces^1^																						*1 *				*2 *			
Total of positive isolates in rooms	**8**	**1**	**0**			**2**	**0**	**6**	**0**	**1**	**0**	**1**	**3**	**0**	**0**	**0**	**0**	**0**				**2**	**1**	**0**		**2**	**0**		

Number of environmental isolates in common areas																													
High drawer chests		0	0			*1 *	*1 *	*1 *	0	*1 *	0	0	*1 *	0	0	0	0	0	0	0	0	*1 *	*1 *	0	0	0	0	0	0
Handles, counter tops and nurse station desk		*1 *	0			0	*1 *	*1 *	0	0	0	0	0	0	0	0	0	0	0	0	0	*1 *	0	0	0	0	0	0	0
Automated medication dispensing system		*1 *	0			*2 *	*1 *	*1 *	0	0	0	0	0	0	0	0	0	0	0	0	0	*1 *	0	0	*1 *	*1 *	0	0	0
Washbasins and taps		0	0			0	0	0	0	0	0	0	0	0	0	0	0	0	0	0	0	0	0	0	0	0	0	0	0
Other objects and surfaces^2^		*1 *											*1 *		*1 *							*1 *				*1 *			
Total of positive isolates in common areas		**3**	**0**			**3**	**3**	**3**	**0**	**1**	**0**	**0**	**2**	**0**	**1**	**0**	**0**	**0**	**0**	**0**	**0**	**4**	**1**	**0**	**1**	**2**	**0**	**0**	**0**

^1^Environmental isolates obtained from triple infusion pump (31th week), hypothermia machine (2 positive samples at 35th week).

^2^Environmental isolates obtained from hypothermia machine (11th, 24th, 31th, and 35th weeks), chair (22th week).

Italic font when the number of positives was equal to or greater than 1, for a better comprehension.

**Table 3:** Poisson regression analysis of different factors involved in the outbreak (univariate analysis).

Factor	*β* coefficient	*P*	RR	95%CI RR
Positive in common areas	0.33	0.051	1.40	0.99–1.94
Positive in rooms	0.17	0.048	1.19	1–1.40
Number of carriers	0.06	0.828	1.06	0.62–1.81
Week (continuous data)	−0.01	0.075	0.99	0.97–1
Month (continuous data)	−0.06	0.068	0.94	0.87–1
Quarter (continuous data)	−0.16	0.126	0.85	0.69–1.05
Quarter (categorical data)				
Q3-2009			1	
Q4-2009	−1.18	0.022	0.31	0.11–0.85
Q1-2010	−0.15	0.688	0.86	0.41–1.78
Q2-2010	−0.49	0.234	0.62	0.28–1.37
Q3-2010	−1.10	0.033	0.33	0.12–0.92

Univariate regression model with the number of infected subjects as the dependent variable, and the following independent variables: the number of positive samples in common areas, the number of positive samples in rooms, the number of carriers, and time: weeks, months, and quarters (Q).

## Abbreviations

MDR: multidrug-resistant
PCR: protein chain reaction
ICU: intensive care unit
UPGMA: unweighted pair group method with arithmetic averages.
